# Supplementary material for: Breast Cancer Molecular Subtypes Differentially Express Gluconeogenic Rate-Limiting Enzymes—Obesity as a Crucial Player
Source: Cancers (Basel). 2023 Oct 11;15(20):4936. doi: 10.3390/cancers15204936 (PMC10605212; doi:10.3390/cancers15204936)
Supplement: Supplementary file 1 [file cancers-15-04936-s001.zip › Supplementary material - Figure S1.pdf]

Supplementary material – Figure S1

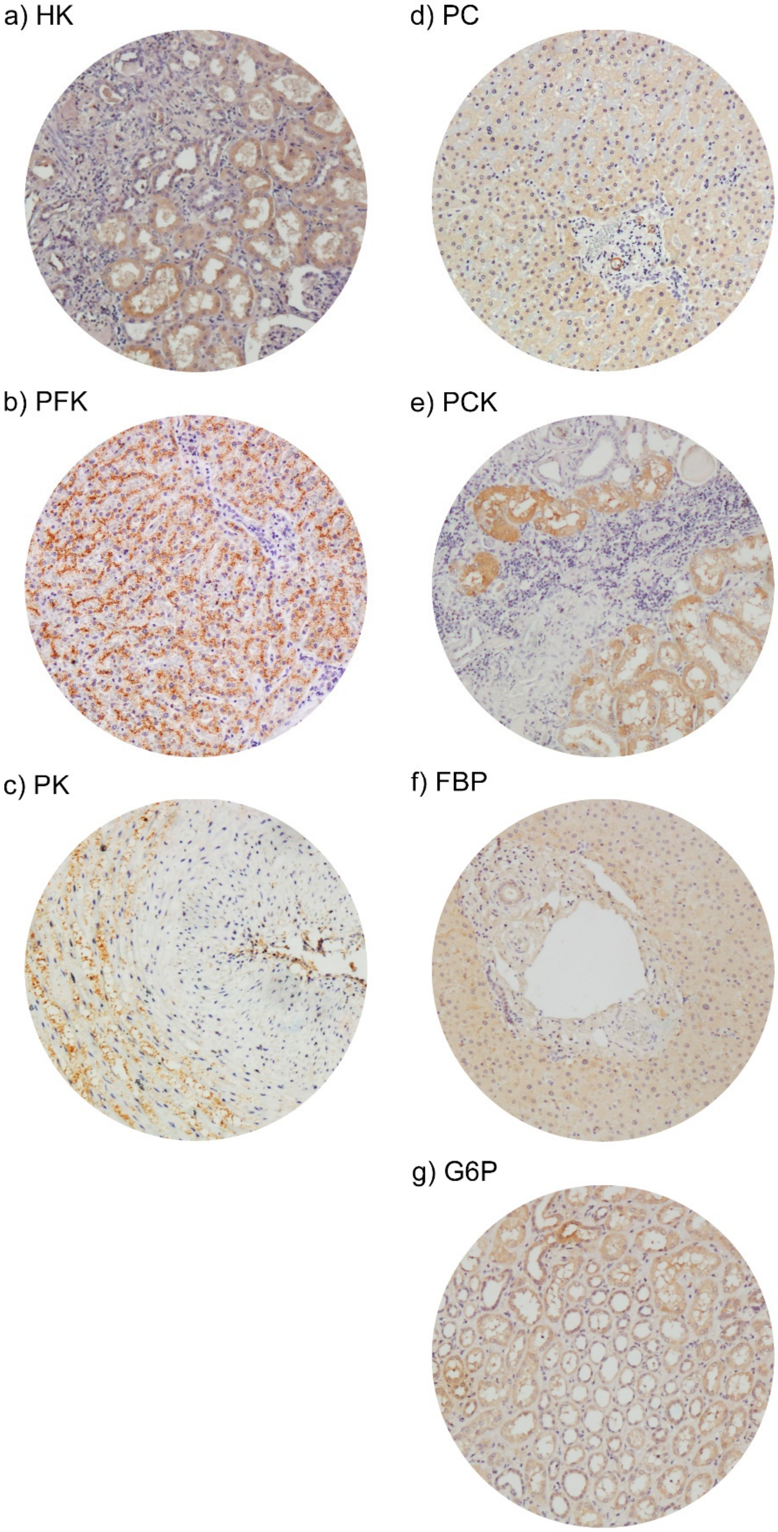

Figure S1. Representative images of positive controls of each assay. a) Kidney, HK – Hexokinase; b) Liver, PFK – Phosphofructokinase; c) Umbilical cord, PK – Pyruvate kinase; d) Liver, PC – Pyruvate carboxylase; e) Kidney, PCK – Phosphoenolpyruvate carboxykinase; f) Liver, FBP – Fructose bisphosphatase; g) Kidney, G6P – Glucose-6-phosphatase.
