# Supplementary material for: Breast Cancer Molecular Subtypes Differentially Express Gluconeogenic Rate-Limiting Enzymes—Obesity as a Crucial Player
Source: Cancers (Basel). 2023 Oct 11;15(20):4936. doi: 10.3390/cancers15204936 (PMC10605212; doi:10.3390/cancers15204936)
Supplement: Supplementary file 1 [file cancers-15-04936-s001.zip › Supplementary material - Figure S2.pdf]

Supplementary material – Figure S2

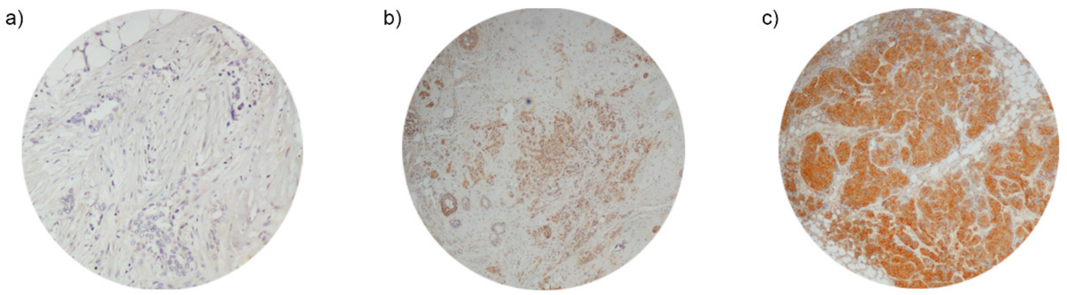

Figure S2. Representative images of expression patterns for a) no expression; b) low expression; and c) high expression, in the example case of pyruvate kinase. Amplification: 100X.
