# Supplementary material for: Breast Cancer Molecular Subtypes Differentially Express Gluconeogenic Rate-Limiting Enzymes—Obesity as a Crucial Player
Source: Cancers (Basel). 2023 Oct 11;15(20):4936. doi: 10.3390/cancers15204936 (PMC10605212; doi:10.3390/cancers15204936)
Supplement: Supplementary file 1 [file cancers-15-04936-s001.zip › Supplementary material - Table S1.pdf]

Supplemental Material – Table S1

Table S1. Immunohistochemistry specifications.

Enzyme	Peroxidase blockage	Blocking solution	Primary antibody		Secondary antibody		Positive Control
			Ref./Man.	Dilution	Ref./ Man.	Dilution	
HK	30min@RT 4% H_2O_2 Methanol	1h@37ºC 0.2% BSA. 10% NSS in PBS	ab104836/ abcam	1:300	ab97021/ abcam	1:200	Kidney
PFK	30min@RT 4% H_2O_2 in PBS	1h@37ºC 4% BSA. 10% HS in PBS	sc-393713/ Santa Cruz Biotechnology	1:400	ab97021/ abcam	1:600	Liver
PK	30min@RT 4% H_2O_2 in PBS	1h@37ºC 4% BSA. 10% HS in PBS	sc-365684/ Santa Cruz Biotechnology	1:400	ab97021/ abcam	1:600	Umbilical cord
PC	30min@RT 4% H_2O_2 in PBS	1h@37ºC 4% BSA. 10% HS in PBS	sc-365673/ Santa Cruz Biotechnology	1:300	ab97021/ abcam	1:400	Liver
PCK	30min@RT 4% H_2O_2 in PBS	1h@37ºC 4% BSA. 10% HS in PBS	sc-271029/ Santa Cruz Biotechnology	1:200	ab97021/ abcam	1:200	Kidney
FBP	30min@RT 4% H_2O_2 in PBS	1h@37ºC 4% BSA. 10% HS in PBS	ab196556/ abcam	1:200	ab97021/ abcam	1:200	Liver
G6P	30min@RT 4% H_2O_2 Methanol	1h@37ºC 4% BSA. 10% HS in PBS	ab243319/ abcam	1:2500	ab97021/ abcam	1:200	Kidney

Footnotes: Ref. – Reference; Man. – Manufacturer; HK – Hexokinase; PFK – Phosphofructokinase; PK – Pyruvate Kinase; PC – Pyruvate Carboxylase; PCK – Phosphoenolpyruvate Carboxykinase; FBP – Fructose-1,6-bisphosphatase; G6P – Glucose-6-Phosphatase; PBS – Phosphate-buffered saline; RT – Room temperature; H_2O_2 – Hydrogen Peroxide; BSA – Bovine Serum Albumin; NSS – Normal Swine Serum; HS – Human serum.

Reagents specifications:

H_2O_2 : Cat. 3323; Alfa Aesar, Haverhill, Mass, EUA.

Methanol: Cat. 179337-2.5L, Merck, Darmstadt, Germany.

PBS: NaCl (Merck, Darmstadt, Germany): 137 mM; KCl (Merck, Darmstadt, Germany): 2.7 mM; Na2HPO4 (Merck, Darmstadt, Germany): 10 mM; KH2PO4 (Merck, Darmstadt, Germany): 1.8 mM in water.

BSA: Cat. A23016, Sigma, Darmstadt, Germany.

NSS: Cat. S-4000-20, Vector laboratories, Newark, NJ, USA.

Antibodies: Santa Cruz Biotechnology, Texas, USA; abcam, Cambridge, United Kingdom.

Hematoxylin: Cat. 72704; Thermo Fisher Scientific, Waltham, Mass, EUA.
