# Supplementary material for: Breast Cancer Molecular Subtypes Differentially Express Gluconeogenic Rate-Limiting Enzymes—Obesity as a Crucial Player
Source: Cancers (Basel). 2023 Oct 11;15(20):4936. doi: 10.3390/cancers15204936 (PMC10605212; doi:10.3390/cancers15204936)
Supplement: Supplementary file 1 [file cancers-15-04936-s001.zip › Supplementary material - Table S2.pdf]

Supplementary material – Table S2

Table S2. Demographic and clinicopathological information cancer associated, from the patients included in the study stratified by (A) body mass index (BMI), molecular subtype (MS), and (B) BMI and molecular subtype (mBMI).

(A)

	Normal weight (18.5 – 24.9 Kg/m^2)	Obese (> 30.0 Kg/m^2)	Total	p-value	Luminal A	Luminal B	Triple Negative	HER2+	Total	p-value
n (%)	32 (51.6%)	30 (48.4%)	62 (100.0%)		16 (25.8%)	16 (25.8%)	16 (25.8%)	14 (22.6%)	62 (100%)	
**Age at diagnosis**										
(Mean ± SD)	56.88 ± 11.15	58.13 ± 9.98	57.48 ± 10.53	0.480	53.44 ± 8.76	58.38 ± 9.11	57.19 ± 13.07	61.43 ± 10.06	57.48 ± 10.53	0.218
**Topographic localization**										
CQ	2 (6.3%)	1 (3.3%)	3 (4.8%)	0.680	0 (0.0%)	3 (18.7%)	0 (0.0%)	0 (0.0%)	3 (4.8%)	0.052
IOQ	1 (3.1%)	3 (10.0%)	4 (6.5%)		0 (0.0%)	1 (6.3%)	2 (12.5%)	1 (7.1%)	4 (6.5%)	
SOQ	13 (40.6%)	8 (26.7%)	21 (33.9%)		5 (31.2%)	1 (6.3%)	9 (56.3%)	6 (42.9%)	21 (33.9%)	
SIQ	2 (6.3%)	2 (6.7%)	4 (6.5%)		0 (0.0%)	2 (12.5%)	1 (6.3%)	1 (7.1%)	4 (6.5%)	
Multiple	12 (37.5%)	15 (50.0%)	27 (43.5%)		11 (68.8%)	8 (50.0%)	3 (18.7%)	5 (35.7%)	27 (43.5%)	
Other	2 (6.2%)	1 (3.3%)	3 (4.8%)		0 (0.0%)	1 (6.3%)	1 (6.3%)	1 (7.1%)	3 (4.8%)	
**Laterality**										
Right	17 (53.1%)	15 (50.0%)	32 (51.6%)	0.806	10 (62.5%)	7 (43.8%)	7 (43.8%)	8 (57.1%)	32 (51.6%)	0.632
Left	15 (46.9%)	15 (50.0%)	30 (48.4%)		6 (37.5%)	9 (56.2%)	9 (56.2%)	6 (42.9%)	30 (48.4%)	
**Pathological stage**										
Stage I	16 (50.0%)	20 (69.0%)	36 (59.0%)	0.321	11 (68.8%)	14 (87.5%)	6 (40.0%)	5 (35.7%)	36 (59.0%)	**0.037***
Stage II	14 (43.8%)	8 (27.6%)	22 (36.1%)		5 (31.2%)	2 (12.5%)	8 (53.3%)	7 (50.0%)	22 (36.1%)	
Stage II	2 (6.2%)	1 (3.4%)	3 (4.9%)		0 (0.0%)	0 (0.0%)	1 (6.7%)	2 (14.3%)	3 (4.9%)	
**Histological grade**										
Grade II	17 (53.1%)	20 (66.7%)	37 (59.7%)	0.227	16 (100.0%)	16 (100.0%)	2 (12.5%)	3 (21.4%)	37 (59.7%)	**<0.001***
Grade III	15 (46.9%)	10 (33.3%)	25 (40.3%)		0 (0.0%)	0 (0.0%)	14 (87.5%)	11 (78.6%)	25 (40.3%)	
**Carcinoma *in situ***										
Presence	11 (34.4%)	13 (43.3%)	24 (38.7%)	0.469	9 (56.3%)	4 (25.0%)	5 (31.3%)	6 (42.9%)	24 (38.7%)	0.282
Size. Cm (Mean ± SD)	1.16 ± 2.02	1.55 ± 2.91	1.35 ± 2.47	0.649	1.70 ± 1.99	0.99 ± 1.95	1.61 ± 3.79	1.04 ± 1.63	1.35 ± 2.47	0.798
Extensive	9 (28.1%)	11 (36.7%)	20 (32.3%)	0.472	8 (50.0%)	4 (25.0%)	4 (25.0%)	4 (28.6%)	20 (32.3%)	0.367
Microcalcifications	14 (43.8%)	14 (46.7%)	28 (45.2%)	0.818	9 (56.3%)	9 (56.3%)	4 (25.0%)	6 (42.9%)	28 (45.2%)	0.236

Necrosis	20 (62.5%)	19 (63.3%)	39 (62.9%)	0.946	10 (62.5%)	9 (56.3%)	7 (43.8%)	13 (92.9%)	39 (62.9%)	**0.042***
**Invasive carcinoma**										
Size. Cm (Mean ± SD)	1.94 ± 0.71	1.57 ± 0.75	1.76 ± 0.75	0.465	1.54 ± 0.34	1.58 ± 0.45	1.97 ± 0.78	1.97 ± 1.16	1.76 ± 0.75	0.197
Multifocality	7 (21.9%)	7 (23.3%)	14 (22.6%)	0.891	5 (31.3%)	3 (18.8%)	2 (12.5%)	4 (28.6%)	14 (22.6%)	0.564
Invasion	11 (34.4%)	8 (26.7%)	19 (30.6%)	0.511	5 (31.3%)	6 (37.5%)	3 (18.8%)	5 (35.7%)	19 (30.6%)	0.661
**Metastatic nodes**										
0	20 (62.5%)	21 (70.0%)	41 (66.2%)	0.364	9 (56.3%)	11 (68.8%)	13 (81.3%)	8 (57.1%)	41 (66.2%)	0.144
1-3	10 (31.3%)	8 (26.7%)	18 (29.0%)		7 (43.7%)	5 (31.2%)	2 (12.5%)	4 (28.6%)	18 (29.0%)	
4-9	0 (0.0%)	1 (3.3%)	1 (1.6%)		0 (0.0%)	0 (0.0%)	1 (6.2%)	0 (0.0%)	1 (1.6%)	
>10	2 (6.2%)	0 (0.0%)	2 (3.2%)		0 (0.0%)	0 (0.0%)	0 (0.0%)	2 (14.3%)	2 (3.2%)	
Size cm (Mean ± SD) (Larger metastasis)	12.66 ± 33.16	10.23 ± 30.11	11.48 ± 31.49	0.764	18.83 ± 39.78	24.77 ± 44.26	0.54 ± 1.22	0.41 ± 0.64	11.48 ± 31.49	0.056

(B)

	Luminal A Normal weight	Luminal A Obese	Luminal B Normal weight	Luminal B Obese	Triple Negative Normal weight	Triple negative Obese	HER2+ Normal weight	HER2+ Obese	Total	p-value
n (%)	8 (12.9%)	8 (12.9%)	8 (12.9%)	8 (12.9%)	8 (12.9%)	8 (12.9%)	8 (12.9%)	6 (9.7%)	62 (100%)	
**Age at diagnosis** (Mean ± SD)	52.13 ± 8.71	54.75 ± 9.19	59.25 ± 10.51	57.50 ± 8.09	56.88 ± 14.93	57.50 ± 11.95	59.25 ± 10.24	64.33 ± 10.09	57.48 ± 10.53	0.604
**Topographic localization**										
CQ	0 (0.0%)	0 (0.0%)	2 (25.0%)	1 (12.5%)	0 (0.0%)	0 (0.0%)	0 (0.0%)	0 (0.0%)	3 (4.8%)	0.075
IOQ	0 (0.0%)	0 (0.0%)	0 (0.0%)	1 (12.5%)	0 (0.0%)	2 (25.0%)	1 (12.5%)	0 (0.0%)	4 (6.5%)	
SOQ	3 (37.5%)	2 (25.0%)	0 (0.0%)	1 (12.5%)	4 (50.0%)	5 (62.5%)	6 (75.0%)	0 (0.0%)	21 (33.9%)	
SIQ	0 (0.0%)	0 (0.0%)	1 (12.5%)	1 (12.5%)	1 (12.5%)	0 (0.0%)	0 (0.0%)	1 (16.7%)	4 (6.5%)	
Multiple	5 (62.5%)	6 (75.0%)	4 (50.0%)	4 50.0%)	2 (25.0%)	1 (12.5%)	1 (12.5%)	4 (66.7%)	27 (43.5%)	
Other	0 (0.0%)	0 (0.0%)	1 (12.5%)	0 (0.0%)	1 (12.5%)	0 (0.0%)	0 (0.0%)	1 (16.7%)	3 (4.8%)	
**Laterality**										
Right	4 (50.0%)	6 (75.0%)	3 (37.5%)	4 (50.0%)	5 (62.5%)	2 (25.0%)	5 (62.5%)	3 (50.0%)	32 (51.6%)	0.606
Left	4 (50.0%)	2 (25.0%)	5 (62.5%)	4 (50.0%)	3 (37.5%)	6 (75.0%)	3 (37.5%)	3 (50.0%)	30 (48.4%)	
**Pathological stage**										
Stage I	6 (75.0%)	5 (62.5%)	6 (75.0%)	8 (100.0%)	3 (37.5%)	3 (42.9%)	1 (12.5%)	4 (66.7%)	36 (59.0%)	0.051

Stage II	2 (25.0%)	3 (37.5%)	2 (25.0%)	0 (0.0%)	5 (62.5%)	3 (42.9%)	5 (62.5%)	2 (33.3%)	22 (36.1%)	
Stage II	0 (0.0%)	0 (0.0%)	0 (0.0%)	0 (0.0%)	0 (0.0%)	1 (14.3%)	2 (25.0%)	0 (0.0%)	3 (4.9%)	
**Histological grade**										
Grade II	8 (100.0%)	8 (100.0%)	8 (100.0%)	8 (100.0%)	0 (0.0%)	2 (25.0%)	1 (12.5%)	2 (33.3%)	37 (59.7%)	<**0.001***
Grade III	0 (0.0%)	0 (0.0%)	0 (0.0%)	0 (0.0%)	8 (100.0%)	6 (75.0%)	7 (87.5%)	4 (66.7%)	25 (40.3%)	
**Carcinoma *in situ***										
Presence	4 (50.0%)	5 (62.5%)	3 (37.5%)	1 (12.5%)	1 (12.5%)	4 (50.0%)	3 (37.5%)	3 (50.0%)	24 (38.7%)	0.357
Size. cm (Mean ± SD)	1.79 ± 2.48	1.61 ± 1.53	1.69 ± 2.52	0.30 ± 0.85	0.33 ± 0.92	2.9 ± 5.12	0.83 ± 1.73	1.33 ± 1.60	1.35 ± 2.47	0.463
Extensive	4 (50.0%)	4 (50.0%)	3 (37.5%)	1 (12.5%)	0 (0.0%)	4 (50.0%)	2 (25.0%)	2 (33.3%)	20 (32.3%)	0.253
Microcalcifications	4 (50.0%)	5 (62.5%)	6 (75.0%)	3 (37.5%)	1 (12.5%)	3 (37.5%)	3 (37.5%)	3 (50.0%)	28 (45.2%)	0.333
Necrosis	5 (62.5%)	5 (62.5%)	5 (62.5%)	4 (50.0%)	2 (25.0%)	5 (62.5%)	8 (100.0%)	5 (83.3%)	39 (62.9%)	0.126
**Invasive carcinoma**										
Size. cm (Mean ± SD)	1.56 ± 0.28	1.53 ± 0.40	1.74 ± 0.50	1.41 ± 0.36	2.2 ± 0.60	1.74 ± 0.92	2.25 ± 1.07	1.60 ± 1.27	1.76 ± 0.75	0.210
Multifocality	3 (37.5%)	2 (25.0%)	1 (12.5%)	2 (25.0%)	0 (0.0%)	2 (25.0%)	3 (37.5%)	1 (16.7%)	14 (22.6%)	0.656
Invasion	3 (37.5%)	2 (25.0%)	3 (37.5%)	3 (37.5%)	1 (12.5%)	2 (25.0%)	4 (50.0%)	1 (16.7%)	19 (30.6%)	0.783
**Metastatic nodes**										
0	5 (62.5%)	4 (50.0%)	6 (75.0%)	5 (62.5%)	7 (87.5%)	6 (75.0%)	2 (25.0%)	6 (100.0%)	41 (66.2%)	
1-3	3 (37.5%)	4 (50.0%)	2 (25.0%)	3 (37.5%)	1 (12.5%)	1 (12.5%)	4 (50.0%)	0 (0,0%)	18 (29.0%)	0.082
4-9	0 (0,0%)	0 (0,0%)	0 (0,0%)	0 (0,0%)	0 (0,0%)	1 (12.5%)	0 (0,0%)	0 (0,0%)	1 (1.6%)	
>10	0 (0,0%)	0 (0,0%)	0 (0,0%)	0 (0,0%)	0 (0,0%)	0 (0,0%)	2 (25.0%)	0 (0,0%)	2 (3.2%)	
Size cm (Mean ± SD) (Larger metastasis)	24.93 ± 45.72	12.74 ± 34.86	24.75 ± 45.83	24.79 ± 45.81	0.24 ± 0.67	0.84 ± 1.59	0.73 ± 0.70	0.00 ± 0.00	11.48 ± 31.49	0.340

Legend: SD – Standard Deviation; CQ – Central Quadrant; IOQ – Inferior Outer Quadrant; SOQ – Superior Outer Quadrant; SIQ – Superior Inner Quadrant; Statistical significance highlighted in bold (p-value<0.05).
