# Supplementary material for: Breast Cancer Molecular Subtypes Differentially Express Gluconeogenic Rate-Limiting Enzymes—Obesity as a Crucial Player
Source: Cancers (Basel). 2023 Oct 11;15(20):4936. doi: 10.3390/cancers15204936 (PMC10605212; doi:10.3390/cancers15204936)
Supplement: Supplementary file 1 [file cancers-15-04936-s001.zip › Supplementary material - Table S3.pdf]

Supplementary material – Table S3

Table S3. Pathological information of obesity associated pathologies – diabetes, hypertension, and dyslipidemia – from the patients included in the study stratified by (A) body mass index (BMI), molecular subtype (MS), and (B) BMI and molecular subtype (mBMI).

(A)

	Normal weight (18.5 – 24.9 Kg/m^2)	Obese (> 30.0 Kg/m^2)	Total	p-value	Luminal A	Luminal B	Triple Negative	HER2+	Total	p-value
n (%)	32 (51.6%)	30 (48.4%)	62 (100.0%)		16 (25.8%)	16 (25.8%)	16 (25.8%)	14 (22.6%)	62 (100%)	
**Diabetes**										
Yes	3 (9.4%%)	4 (13.3%)	7 (11.3%)		1 (6.3%)	2 (12.5%)	2 (12.5%)	2 (14.3%)	7 (11.3%)	
No	29 (90.6%)	25 (83.3%)	54 (87.1%)	0.589	15 (93.7%)	14 (87.5%)	14 (87.5%)	11 (78.6%)	54 (87.1%)	0.883
Missing data	0 (0.0%)	1 (3.4%)	1 (1.6%)		0 (0.0%)	0 (0.0%)	0 (0.0%)	1 (7.1%)	1 (1.6%)	
**Hypertension**										
Yes	8 (25.0%)	17 (56.6%)	25 (40.3%)		6 (37.5%)	7 (43.8%)	6 (37.5%)	6 (42.9%)	25 (40.3%)	
No	24 (75.0%)	12 (40.0%)	36 (58.1%)	**0.008***	10 (62.5%)	9 (56.2%)	10 (62.5%)	7 (50.0%)	36 (58.1%)	0.949
Missing data	0 (0.0%)	1 (3.4%)	1 (1.6%)		0 (0.0%)	0 (0.0%)	0 (0.0%)	1 (7.1%)	1 (1.6%)	
**Dyslipidemia**										
Yes	9 (28.1%)	13 (43.3%)	22 (35.5%)		5 (31.2%)	8 (50.0%)	4 (25.0%)	5 (35.7%)	22 (35.5%)	
No	23 (71.9%)	16 (53.3%)	39 (62.9%)	0.175	11 (68.8%)	8 (50.0%)	12 (75.0%)	8 (57.2%)	39 (62.9%)	0.495
Missing data	0 (0.0%)	1 (3.4%)	1 (1.6%)		0 (0.0%)	0 (0.0%)	0 (0.0%)	1 (7.1%)	1 (1.6%)	

(B)

	Luminal A Normal weight	Luminal A Obese	Luminal B Normal weight	Luminal B Obese	Triple Negative Normal weight	Triple negative Obese	HER2+ Normal weight	HER2+ Obese	Total	p-value
n (%)	8 (12.9%)	8 (12.9%)	8 (12.9%)	8 (12.9%)	8 (12.9%)	8 (12.9%)	8 (12.9%)	6 (9.7%)	62 (100%)	
**Diabetes**										
Yes	1 (12.5%)	0 (0.0%)	1 (12.5%)	1 (12.5%)	0 (0.0%)	2 (25.0%)	1 (12.5%)	1 (16.7%)	7 (11.3%)	
No	7 (87.5%)	8 (100.0%)	7 (87.5%)	7 (87.5%)	8 (100.0%	6 (75.0%)	7 (87.5%)	4 (66.6%)	54 (87.1%)	0.791
Missing data	0 (0.0%)	0 (0.0%)	0 (0.0%)	0 (0.0%)	0 (0.0%)	0 (0.0%)	0 (0.0%)	1 (16.7%)	1 (1.6%)	
**Hypertension**										
Yes	2 (25.0%)	4 (50.0%)	3 (37.5%)	4 (50.0%)	2 (25.0%)	4 (50.0%)	1 (12.5%)	5 (83.3%)	25 (40.3%)	
No	6 (75.0%)	4 (50.0%)	5 (62.5%)	4 (50.0%)	6 (75.0%)	4 (50.0%)	7 (87.5%)	0 (0.0%)	36 (58.1%)	0.088
Missing data	0 (0.0%)	0 (0.0%)	0 (0.0%)	0 (0.0%)	0 (0.0%)	0 (0.0%)	0 (0.0%)	1 (16.7%)	1 (1.6%)	
**Dyslipidemia**										
Yes	1 (12.5%)	4 (50.0%)	4 (50.0%)	4 (50.0%)	1 (12.5%)	3 (37.5%)	3 (37.5%)	2 (33.3%)	22 (35.5%)	
No	7 (87.5%)	4 (50.0%)	4 (50.0%)	4 (50.0%)	7 (87.5%)	5 (62.5%)	5 (62.5%)	3 (50.0%)	39 (62.9%)	0.549
Missing data	0 (0.0%)	0 (0.0%)	0 (0.0%)	0 (0.0%)	0 (0.0%)	0 (0.0%)	0 (0.0%)	1 (16.7%)	1 (1.6%)	

Legend: Statistical significance highlighted in bold (p-value<0.05).
